# Epithelial–Mesenchymal Transition (EMT) Induced by TNF-α Requires AKT/GSK-3β-Mediated Stabilization of Snail in Colorectal Cancer

**DOI:** 10.1371/journal.pone.0056664

**Published:** 2013-02-19

**Authors:** Hao Wang, Hong-Sheng Wang, Bin-Hua Zhou, Cui-Lin Li, Fan Zhang, Xian-Feng Wang, Ge Zhang, Xian-Zhang Bu, Shao-Hui Cai, Jun Du

**Affiliations:** 1 Department of Microbial and Biochemical Pharmacy, School of Pharmaceutical Sciences, Sun Yat-sen University, Guangzhou, China; 2 Department of Pharmacology, College of Pharmacy, Jinan University, Guangzhou, China; H. Lee Moffitt Cancer Center & Research Institute, United States of America

## Abstract

Chronic inflammation-promoted metastasis has been considered as a major challenge in cancer therapy. Pro-inflammatory cytokine TNFα can induce cancer invasion and metastasis associated with epithelial–mesenchymal transition (EMT). However, the underlying mechanisms are not entirely clear. In this study, we showed that TNFα induces EMT in human HCT116 cells and thereby promotes colorectal cancer (CRC) invasion and metastasis. TNFα-induced EMT was characterized by acquiring mesenchymal spindle-like morphology and increasing the expression of N-cadherin and fibronectin with a concomitant decrease of E-cadherin and Zona occludin-1(ZO-1). TNFα treatment also increased the expression of transcription factor Snail, but not Slug, ZEB1 and Twist. Overexpression of Snail induced a switch from E-cadherin to N-cadherin expression in HCT116 cells, which is a characteristic of EMT. Conversely, knockdown of Snail significantly attenuated TNFα-induced EMT in HCT116 cells, suggesting that Snail plays a crucial role in TNFα-induced EMT. Interestingly, exposure to TNFα rapidly increased Snail protein expression and Snail nuclear localization but not mRNA level upregulation. Finally, we demonstrated that TNFα elevated Snail stability by activating AKT pathway and subsequently repressing GSK-3β activity and decreasing the association of Snail with GSK-3β. Knockdown of GSK-3β further verified our finding. Taken together, these results revealed that AKT/GSK-3β-mediated stabilization of Snail is required for TNFα-induced EMT in CRC cells. Our study provides a better understanding of inflammation-induced CRC metastasis.

## Introduction

Chronic inflammation has been identified to be intimately associated with tumorigenesis [1], [2]. Increasing evidences have proved that the inflammatory tumor microenvironment plays a crucial role in tumor development and metastasis [3]. Tumor microenvironment is largely orchestrated by inflammatory cells, which facilitate extracellular matrix breakdown, angiogenesis, and tissue remodeling, thus promote tumor cell motility [4]. Furthermore, tumor cells themselves can secrete proinflammatory cytokines which contribute directly to malignant progression [5]. The complex interactions between the tumor and inflammatory cells mediated by inflammatory cytokines are an essential aspect of the tumor microenvironment [6]. Tumor necrosis factor α (TNFα), a proinflammatory cytokine predominantly produced by macrophages, is a key molecule regulating the inflammatory processes in tumor promotion. Mounting evidences suggested that TNFα mediates many critical processes of tumor progression, including oncogene activation, DNA damage, and tumor metastasis [7].

Epithelial–mesenchymal transition (EMT), an essential phenotypic conversion during embryonic development, tissue remodeling, and wound healing, plays an indispensable role in tumor invasion and metastasis [8]–[10]. EMT is a reversible process that often occurs at the invasive front of many metastatic cancers [11]. EMT can be triggered by different signals received from tumor microenvironment, such as TGFβ, EGF, WNTs and Notch [12]. During the processes of EMT, epithelial cells loss intercellular adhesion, acquire fibroblast-like characteristics and increase migratory and invasive properties [13]. One of the most well-defined features of EMT is the loss of E-cadherin expression [8]. A group of transcription factors, including Snail, Slug, ZEB1, Twist, have been implicated in the control of EMT [14]. Snail, a zinc-finger transcription factor first identified in Drosophila, has been proved as a key EMT regulator [15]. Studies showed that Snail represses E-cadherin transcription by binding to the E-box site in the promoter of E-cadherin [16]–[18]. The roles of Snail in EMT regulation have been reported in many types of cancer such as breast carcinoma, ovarian carcinoma, etc. [14], [18], [19]. Silencing of Snail by stable RNA interference induces the complete mesenchymal to epithelial transition (MET) in MDCK-Snail cells, which associates with the inhibition of invasion [20]. In addition, high expression of Snail also correlates with tumor grade, recurrence, nodal metastasis and poor outcomes in patients [21]–[25]. Several inflammatory mediators such as TGFβ, hypoxia and IL-6 can upregulate Snail and therefore trigger EMT [3]. These findings highlight the importance of the microenvironment in regulation of Snail and in the initiation of EMT.

The colorectal cancer (CRC) is a major worldwide health concern. Most deaths from CRC are due to metastases that are resistant to conventional therapies. EMT is a highly relevant issue to CRC metastasis [26]. However, the role of TNFα in EMT of CRC is rarely investigated and the underlying molecular mechanism remains unclear. Here we showed that TNFα-induced EMT by stabilizing Snail in HCT116 and Caco-2 cells. We also demonstrated that TNFα stabilizes Snail by activating AKT pathway and thereby inhibiting GSK-3β activity and decreasing the association of GSK-3β and Snail.

## Materials and Methods

### Chemicals and Reagents

NF-κB inhibitor BAY11-7082, ERK inhibitor PD98059, p38 MAPK inhibitor SB-203580, PI3K inhibitor LY294002, GSK-3β inhibitor lithium (LiCl) and proteasome inhibitor MG132 were obtained from Sigma-Aldrich (St Louis, MO). Primary antibodies against E-cadherin, Zona occludin-1(ZO-1), Snail, ZEB1, p-GSK-3β (ser9), GSK-3β, p-Akt (Ser473), Akt, and β-catenin were obtained from Cell Signaling Technology (MA, USA). Primary antibody against Histone H2A.X was obtained from Bioworld (Bioworld Technology, Minneapolis, MN, USA). Protein A/G Sepharose and primary antibodies against N-cadherin, ubiquitin, β-actin, α-tubulin were obtained from Santa Cruz Biotechnology (Santa Cruz, CA, USA). Primary antibody to fibronectin was obtained from Boster Biological Engineering. Horseradish peroxidase (HRP)-conjugated secondary antibody, Alexa Fluor 488/594 conjugated secondary antibody, DAPI and lipofectamine 2000 were purchased from Invitrogen (Carlsbad, CA, USA). Recombinant human TNFα protein was bought from PeproTech. PrimeScript® RT reagent Kit and SYBR® Premix Ex Taq™ were products of TaKaRa. E.Z.N.A® HP Total RNA Kit was bought from Omega Bio-Tek (Doraville, USA). Smart pool siRNA against human Snail and GSK-3β were from RIBOBIO.

### Cell Culture

The HCT116 and Caco-2 colorectal carcinoma cell lines were obtained from the Type Culture Collection of the Chinese Academy of Sciences (Shanghai, China). HCT116 cells were maintained in McCoy’5a culture medium (Gibco BRL) supplemented with 10% fetal bovine serum, and Caco-2 cells were cultured in DMEM culture medium (Gibco BRL) supplemented with 10% fetal bovine serum under a humidified 5% CO_2_ atmosphere at 37°C in incubator.

### Transwell Migration and Invasion Assay

Migration and invasion assays were performed in Boyden chambers. The polycarbonate filters (8 µm pore size, Corning) pre-coated with Matrigel Matrix (BD Biosciences) were used for invasion assay, and uncoated filters were used for migration assay. Cells (1×10^5^) in 300 µl medium (containing 0.1% FBS) with or without 20 ng/ml TNFα were seeded in the upper chamber. Then 600 ml medium with 10% FBS was added to the lower chamber and served as a chemotactic agent. After 24 h incubation, for migration, the cells migrated and adhered onto the lower chamber were fixed in 4% paraformaldehyde for 20 min, stained with hematoxylin and counted under upright microscope (5 fields per chamber). For invasion, the cells in the upper chamber were fixed in 4% paraformaldehyde for 20 min. Then the matrigel was mechanically removed from the filter with a cotton swab. The cells adhering to the under-side of the filter were stained with hematoxylin and counted under upright microscope (5 fields per chamber). Each migration and invasion assay was repeated in three independent experiments.

### Gene Over-expression and RNA Interference

The cells were seeded on a 6-well plate (2×10^5^ cells/well) and left in culture until the next day. They were then transfected with 2 µg plasmid vector or 100 pmol siRNA oligomer mixed with lipofectamine 2000 reagent in serum reduced medium according to the manufacturer’s instructions. Medium was changed to complete culture medium 6 h later, and the cells were incubated at 37°C in a CO_2_ incubator for another 24 to 48 h before harvest.

### Quantitative Real-Time PCR

Total mRNA of the cells was extracted after treatment for the indicated time. First strand cDNA synthesis was generated from 500 ng of total RNA. Quantification of target and reference (GAPDH) genes was performed in triplicate on LightCycler® 480 II (Roche, Applied Science). The primers used in each reaction were as follows: E-cadherin forward 5′-TACACTGCCCAGGAGCCAGA-3′ and reverse 5′-TGGCACCAGTGTCCGGATTA-3′; N-cadherin, forward 5′-CGAATGGATGAAAGACCCATCC-3′ and reverse 5′-GGAGCCACTGCCTTCATAGTCAA-3′; Snail, forward 5′- GACCACTATGCCGCGCTCTT-3′ and reverse 5′-TCGCTGTAGTTAGGCTTCCGATT-3′; ZEB1, forward 5′-TACAGAACCCAACTTGAACGTCACA-3′ and reverse 5′- GATTACACCCAGACTGCGTCACA-3′; Twist, forward 5′-GGAGTCCGCAGTCTTACGAG-3′ and reverse 5′-TCTGGAGGACCTGGTAGAGG-3′; Slug, forward 5′-TTCGGACCCACACATTACCT-3′ and reverse 5′-GCAGTGAGGGCAAGAAAAAG-3′; GAPDH, forward 5′- GCACCGTCAAGGCTGAGAAC-3′ and reverse 5′-TGGTGAAGACGCCAGTGGA-3′. After normalized to GAPDH gene, expression levels for each target gene were calculated using the comparative threshold cycle (CT) method. The Δct values were calculated according to the formula Δct = ct (gene of interest)-ct (GAPDH) in correlation analysis, and the 2-ΔΔct was calculated according to the formula ΔΔct = Δct (control group)−Δct (experimental group) for determination of relative. Data are presented as the mean ± standard deviation (SD) from three independent experiments.

### Western Blotting Analysis

The cells were washed three times with ice-cold phosphate buffer solution (PBS) and then lysed in lysis buffer containing 50 mM Tris-HCl (pH 7.6), 150 mM NaCl, 1 mM EDTA, 1% NP-40, 0.5% Na-deoxycholate, 5 µg/ml aprotinin, 5 µg/ml leupeptin, and 1 mM phenylmethylsulfonyl fluoride. Lysates were cleared by centrifugation and denatured by boiling in Laemmli buffer. Equal amounts of protein samples were loaded per well and separated on SDS-polyacrylamide gels, and then electrophoretically transferred onto PVDF membranes. Following blocking with 5% non-fat milk at room temperature for 2 h, membranes were incubated with primary antibodies (1∶1,000 dilution) at 4°C overnight and then incubated with HRP-conjugated secondary antibodies (1∶5,000 dilution) for 2 h at room temperature. Specific immune complexes were detected using Western Blotting Plus Chemiluminescence Reagent (Life Science).

### Immunofluorescence

The cells were cultured on chamber slides, serum starved for 12 h, then exposed to TNFα for the indicated time. Cells were washed three times with PBS, fixed with 4% paraformaldehyde for 20 min and permeabilized with 0.3% Triton X-100 for 10 min. After blocking with goat serum for 2 h at room temperature, cells were incubated with antibodies against E-cadherin, N-cadherin, fibronectin, ZO-1 or Snail (1∶100 dilution) at 4°C overnight. Slides were washed three times with PBS and incubated with Alexa Fluor 488 or Alexa Fluor 594-conjugated secondary antibodies (1∶1,000 dilution) for 1 h at room temperature. Nuclei were stained with DAPI (10 µg/ml) for 10 min. Samples were examined with Confocal Laser Scanning Microscopy (Zeiss) to analyze expression of E-cadherin, N-cadherin, fibronectin, ZO-1 and nuclear translocation of Snail.

### Immunoprecipitation

The cells were washed three time with ice-cold PBS and harvested at 4°C in immunoprecipitation lysis buffer containing 50 mM HEPES, pH 7.5, 150 mM NaCl, 0.5% NP-40, 2 mM EDTA, 10% glycerol, 1 mM Na_3_VO_4_, 1 mM NaF, 1 mM dithiothreitol, 1 mM 4-(2-aminoethyl) benzenesulfonyl fluoride,1 µg/ml leupeptin, 1 µg/ml pepstatin and 1 µg/ml aprotinin. Equal amounts of protein were immunoprecipitated using anti-Snail or anti-GSK-3β antibody, and the immune complexes were bound to protein A/G Sepharose. The beads were washed with lysis buffer and subjected to western blotting with anti-ubiquitin, anti-Snail or anti-GSK-3β antibody.

### Statistical Analysis

Results were expressed as Mean ± SD of three independent experiments unless otherwise specified. Data were analyzed by two-tailed unpaired Student’s t-test between any two groups. One-way ANOVA analysis of variance was used to assess the difference of means among groups. These analyses were performed using GraphPad Prism Software Version 5.0 (GraphPad Software Inc., La Jolla, CA). A P-value of <0.05 was considered statistically significant.

## Results

### TNFα Promotes Migration and Invasion of HCT116 Cells

**Figure 1:**
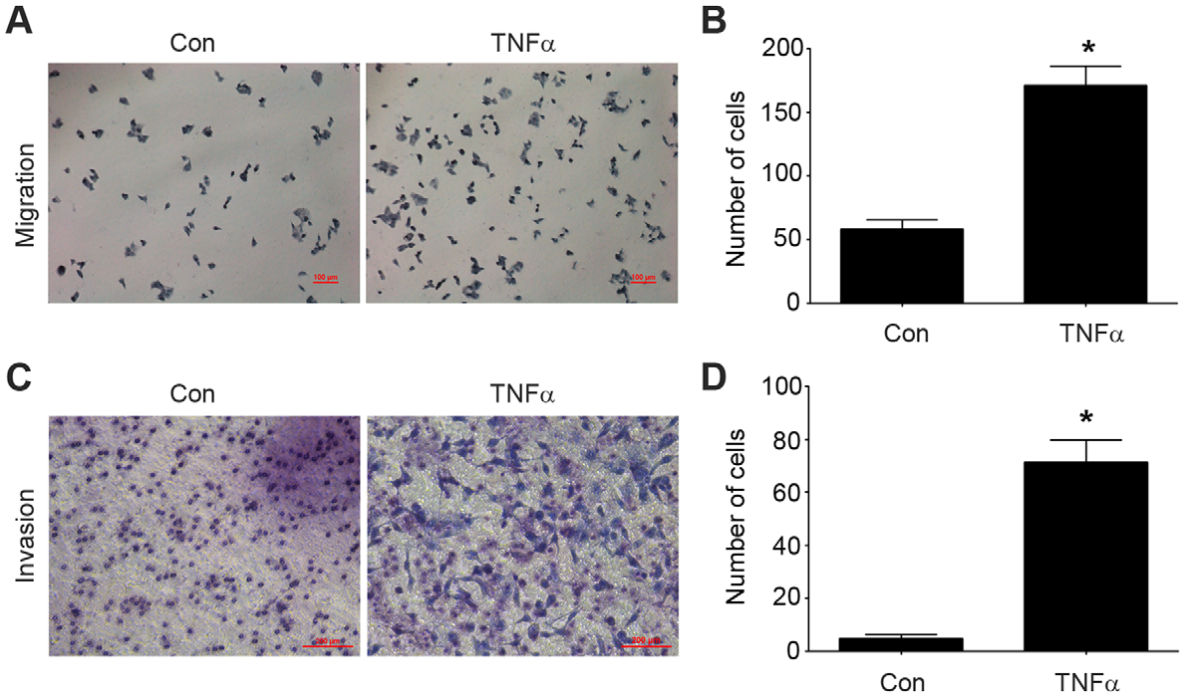
TNFα induces migration and invasion of HCT116 cells. (A) HCT116 cells were allowed to migrate transwell chambers for 24 h in the presence or absence of TNFα (20 ng/ml). After 24 h, the migrated cells were fixed, stained, and photographed. Magnification, 100×. (B) The number of migrated cells. Data represent the average of three independent experiments. (C) After treatment with or without TNFα (20 ng/ml) for 48 h, HCT116 cells that had spread through the matrixgel and into the under-side of the filter were fixed, stained, and photographed. Magnification, 200×. (D) The number of invasive cells. Data represent the average of three independent experiments. *p<0.05 compared with control.

Tumor cells with an aggressive phenotype acquire migratory and invasive capabilities. This will promote the dissemination of tumors cells to distant organs [8]. The migration and invasion abilities of HCT116 cells affected by TNFα were measured by using transwell migration and invasion assays. As shown in Figure 1A and C, TNFα treatment resulted in a significant increase in cell migration and invasion. Compared with control, the number of migrated and invasive cells increased about 4-fold (migration) and 20-fold (invasion) after treatment with TNFα (Fig. 1B and D).

### TNFα Induces EMT in HCT116 Cells

**Figure 2:**
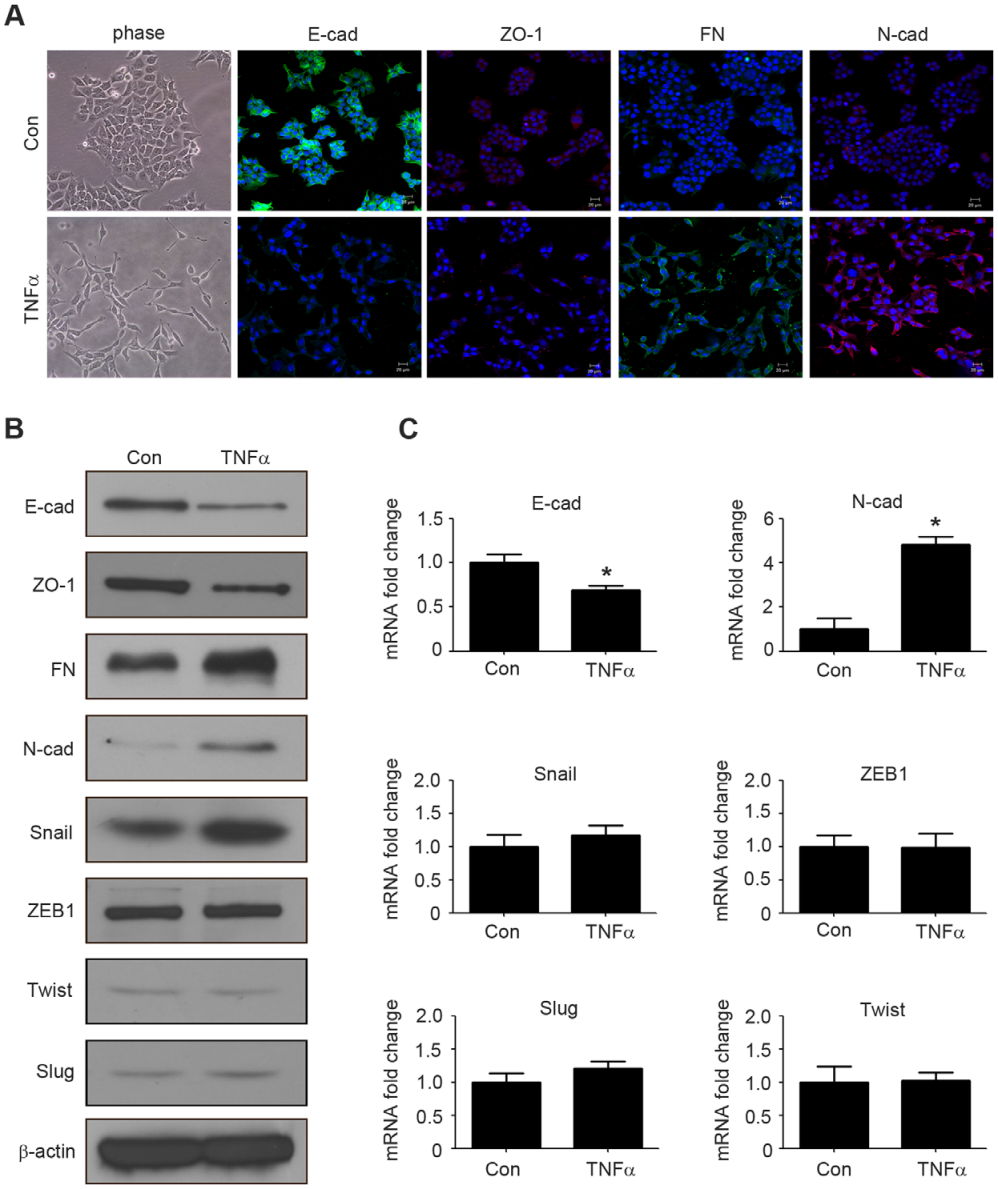
TNFα treatment triggers EMT in HCT116 cells. (A) HCT116 cells were treated with or without TNFα (20 ng/ml) for 4 days. Cell morphological changes associated with EMT are shown in the phase contrast image. Expression of E-cadherin, ZO-1, fibronectin, N-cadherin were analyzed by immunofluorescence staining. Nuclei were visualized with DAPI staining. Scale bars: 20 µm. (B) HCT116 cells were treated with or without TNFα (20 ng/ml) for 4 days, and the expression of E-cadherin, ZO-1, fibronectin, N-cadherin, Snail, ZEB1, Twist, Slug were analyzed by western blotting. β-actin servers as the loading control. (C) HCT116 cells were treated with or without TNFα (20 ng/ml) for 4 days. The mRNA levels of E-cadherin, N-cadherin, Snail, ZEB1, Twist, Slug were analyzed by qRT-PCR. *p<0.05 compared with control.

The increased migration and invasion abilities of tumor cells are reminiscent of the events at EMT, during which, the epithelial makers E-cadhein and ZO-1 are down-regulated, whereas the mesenchymal markers N-cadherin and fibronectin are up-regulated [27]. The EMT of HCT116 cells was observed after stimulation with 20 ng/ml TNFα for 4 days. Cells resulted in a significant change in morphology, from cobblestone morphology to mesenchymal spindle-like and fusiform features (Fig. 2A). Immunofluorescence analysis showed that this morphological change was associated with the down regulation of epithelial characteristics E-cadherin, ZO-1 expression and the upregulation of mesenchymal characteristics fibronectin and N-cadherin expression (Fig. 2A). Similarly, western blotting analysis further confirmed the increasing expression of fibronectin and N-cadherin, and the decreasing expression of E-cadherin and ZO-1 at protein levels (Fig. 2B). Furthermore, qRT-PCR analysis showed that TNFα treatment down-regulated E-cadherin and up-regulated N-cadherin at mRNA levels (Fig. 2C). Collectively, these observations suggested that HCT116 cells had undergone an EMT after treated by TNFα.

### Snail is Crucial for TNFα-mediated EMT

Since transcription factors Snail, ZEB1, Twist and Slug play essential roles in regulating EMT [14], we then investigated whether their expressions were up-regulated in HCT116 cells after treated with TNFα. Compared to untreated cells, TNFα significantly increased Snail protein level, but not mRNA level (Fig. 2B and C). However, TNFα treatment altered neither mRNA nor protein levels of ZEB1, Twist, Slug (Fig. 2B and C).

**Figure 3:**
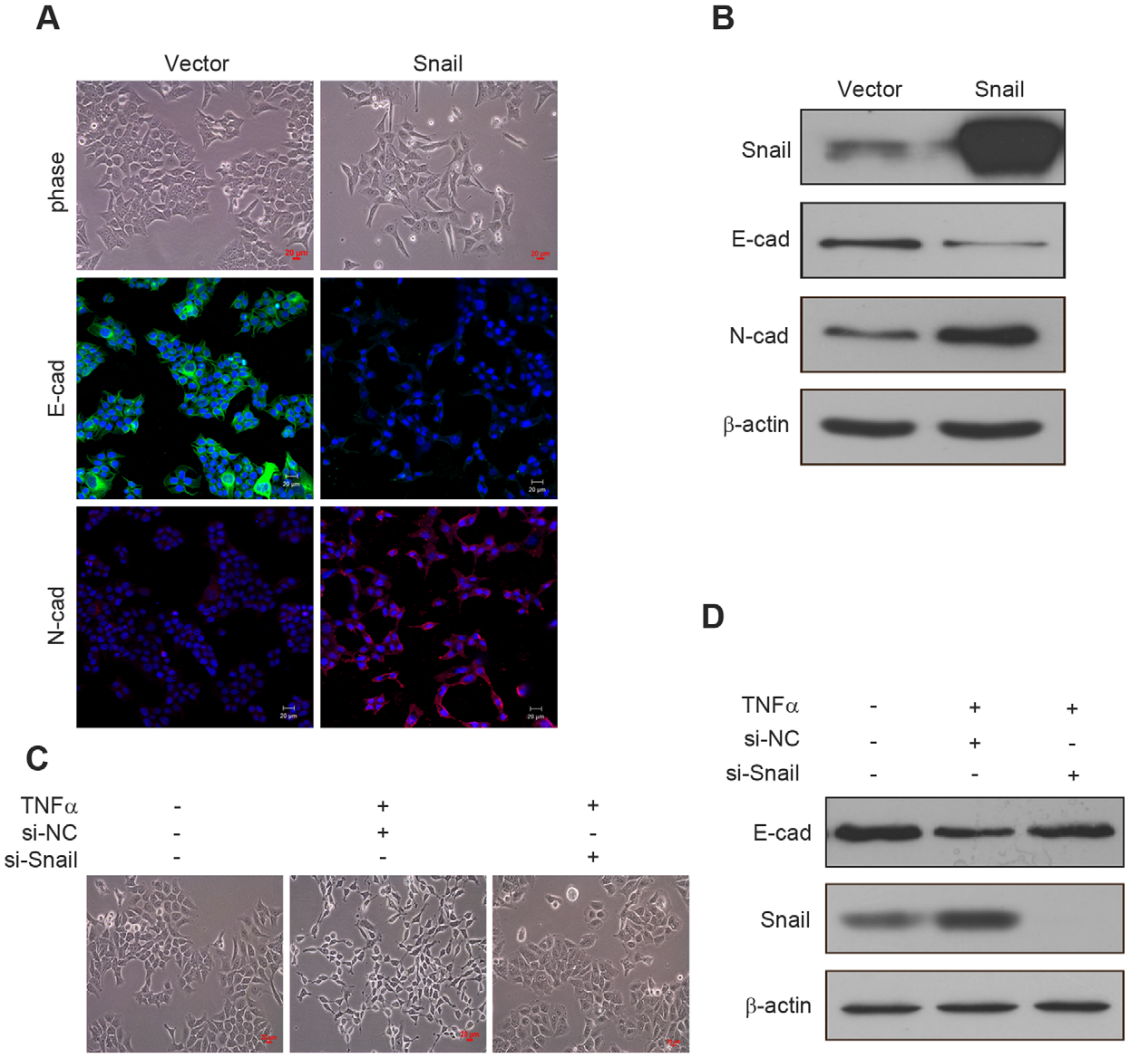
Snail is crucial for TNFα-induced EMT. (A) pcDNA-Snail (Snail) or control vector pcDNA-3.1 (Vector) were expressed in HCT116 cells for 48 h. Cell morphological changes associated with EMT are shown in the phase contrast image. Expression of E-cadherin and N-cadherin were analyzed by immunofluorescence staining. Nuclei were visualized with DAPI staining. Scale bars: 20 µm. (B) The expression of E-cadherin, N-cadherin and Snail from HCT116 cells transfected with pcDNA-Snail or control vector were examined by western blotting. β-actin servers as the loading control. (C) HCT116 cells transfected with Snail specific si-RNA (si-Snail) or negative control si-RNA (si-NC) were stimulated with or without TNFα (20 ng/ml) for 48 h, and the morphologic changes were observed with a phase-contrast microscopy. (D) HCT116 cells transfected with si-Snail or si-NC were stimulated with or without TNFα (20 ng/ml) for 4 days, and the expression of E-cadherin and Snail were detected by western blotting. β-actin servers as the loading control.

We overexpressed Snail to further investigate its roles in TNFα-induced EMT of HCT116 cells. Cells were transfected with pcDNA-Snail and control vector pcDNA-3.1, respectively. Expression of Snail and EMT markers were detected by immunofluorescence and western blotting. The results revealed that increased Snail expression induced EMT-like morphological changes and caused a switch from E-cadherin to N-cadherin expression in HCT116 cells (Fig. 3A and B). These findings demonstrated that ectopic expression of Snail can trigger EMT in HCT116 cells. Based on these observations, we assessed that Snail up-regulation may be crucial for TNFα-induced EMT in HCT116 cells.

We further performed knockdown assays to verify that Snail is a key regulator in TNFα-induced EMT. HCT116 cells were transfected with non-targeting control si-RNA or si-Snail for 24 h, and then treated with TNFα for different time. Morphological changes were observed under a phase contrast microscope. The expressions of Snail and E-cadherin were detected by western blotting. Compared to the control group, the spindle-like morphological changes were not observed upon TNFα addition in si-Snail transfected cells (Fig. 3C). Silencing of Snail also attenuated TNFα-induced down-regulation of E-cadherin, which was not observed in control si-RNA-transfected cells (Fig. 3D). Taken together, these observations demonstrated that Snail is essential for TNFα-induced EMT in HCT116 cells.

### TNFα Regulates Stabilization and Subcellular Localization of Snail

**Figure 4:**
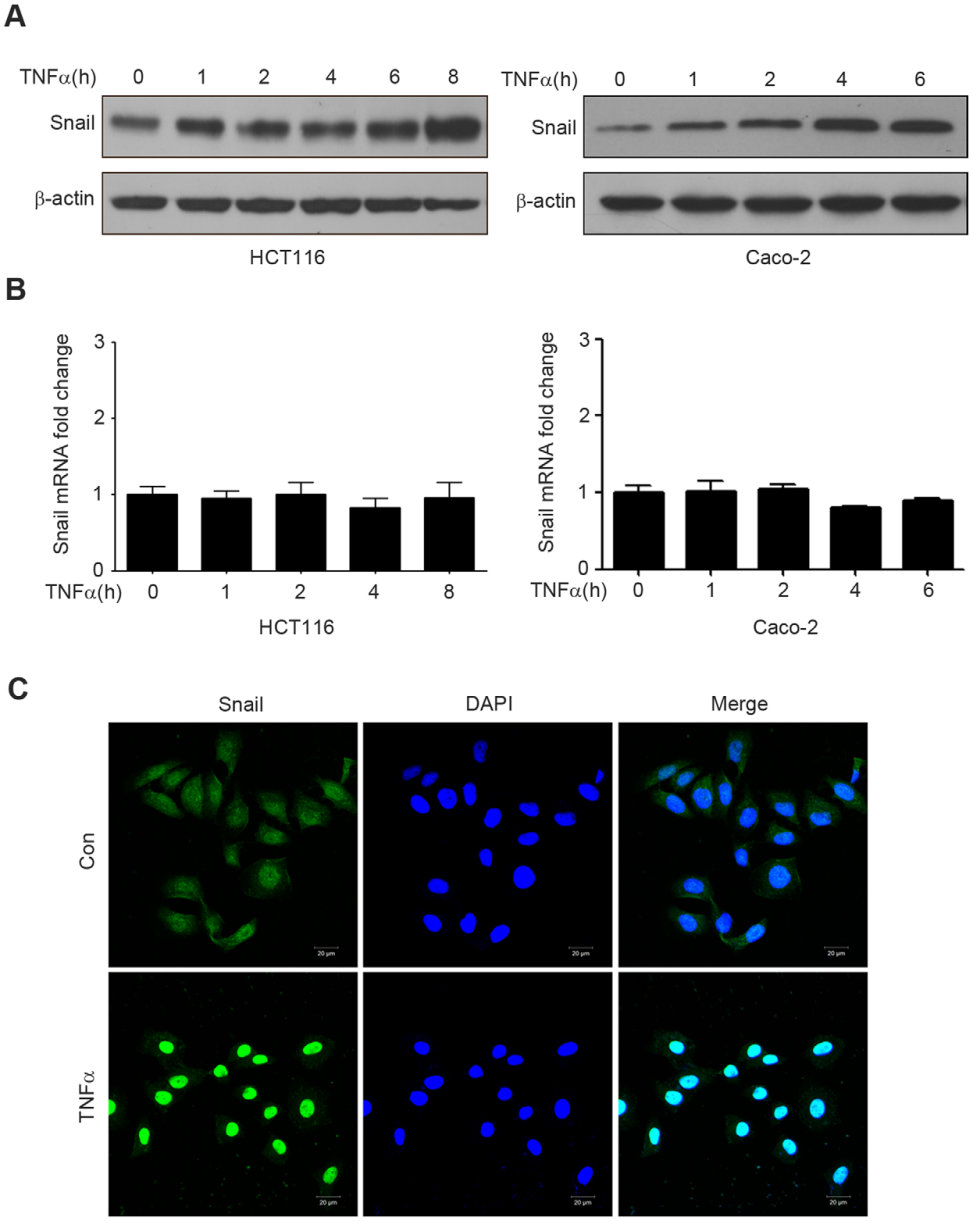
TNFα regulates Snail stabilization and localization. (A–B) HCT116 and Caco-2 cells were treated with TNFα (20 ng/ml) for the times indicated, and the protein (A) and mRNA (B) levels of Snail were examined by western blotting and qRT-PCR respectively; (C) HCT116 cells were treated with or without TNFα (20 ng/ml) for 6 h. After fixation, the cellular location of Snail (green) was examined by immunofluorescence staining and nuclei were stained with DAPI (blue). Scale bars: 20 µm.

We previously found that TNFα increased Snail protein level, but not mRNA level (Fig. 2B and C). This result suggested that up-regulation of Snail by TNFα was occurring at the post-transcriptional level. To further verify this view, HCT116 and Caco-2 cells were treated with TNFα for 0–8 h, and Snail protein and mRNA were detected by western blotting and qRT-PCR, respectively. The results showed that the protein level of Snail was enhanced after 1 h of TNFα stimulation and increased time-dependently (Fig. 4A). However, the mRNA level of Snail did not have a significant change after TNFα treatment (Fig. 4B). These results suggested that the upregulation of Snail by TNFα might be due to protein stabilization.

Since transcription factors can only take effect when they transfer into the nucleus [28], we also determined the nuclear translocation activity of Snail by immunofluorescence. The nuclear localization of Snail was assessed in HCT116 cells stimulated with or without TNFα for 8 h. As shown in Fig. 4C, compared with the control group, TNFα significantly increased the nuclear translocation of Snail.

### TNFα Mediates Snail Stabilization via Activation of AKT and Inhibition of GSK-3β

**Figure 5:**
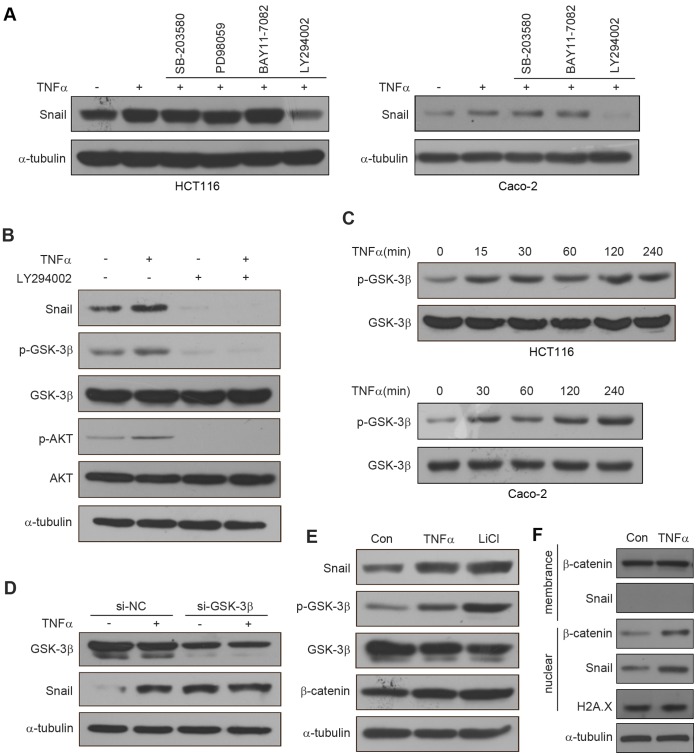
TNFα stabilizes Snail through AKT/GSK3β **pathway.** (A) HCT116 cells were pretreated with SB-203580 (20 µM), PD98059 (20 µM), BAY11-7082(10 µM), LY294002 (20 µM) for 1 h respectively followed by stimulation with TNFα (20 ng/ml) for 6 h. The expression of Snail was examined by western blotting. Caco-2 cells were pretreated with SB-203580 (20 µM), BAY11-7082(10 µM), LY294002 (20 µM) for 1 h respectively followed by stimulation with TNFα (20 ng/ml) for 6 h. The expression of Snail was examined by western blotting. (B) HCT116 cells were pretreated with or without LY294002 (20 µM) for 1 h, followed by stimulation with or without TNFα (20 ng/ml) for 6 h. The expression of Snail and the activation of AKT and GSK-3β were examined by western blotting. (C) HCT116 and Caco-2 cells were treated with TNFα (20 ng/ml) for the times indicated. The expression of pGSK-3β and GSK-3β were examined by western blotting. (D) Control and GSK-3β si-RNA were expressed in HCT116 cells for 42 h, followed treated with or without TNFα (20 ng/ml) for additional 6 h. The expression of Snail and GSK-3β were examined by western blotting. (E) HCT116 cells were treated with TNFα (20 ng/ml) or LiCl (40 mM) for 6 h. The expression of Snail, pGSK-3β, GSK-3β, and β-catenin were analyzed by western blotting. (F) After treated HCT116 cells with or without TNFα (20 ng/ml) for 6 h, Snail and β-catenin located at membrane and nuclear were isolated respectively and then analyzed by western blotting.

To investigate the molecular mechanisms underlying TNFα-mediated Snail stabilization, inhibitors of NF-κB (BAY11-7082), PI3K/AKT (LY294002), MAPK (PD98059), p38 (SB-203580) were used, since TNFα can induce the activation of these pathways. HCT116 and Caco-2 cells were pretreated with inhibitors for 1 h before TNFα stimulation, and then the expression of Snail was determined by western blotting. We found that PI3K/AKT inhibitor (LY294002), but not the others, completely blocked TNFα-stabilized Snail (Fig. 5A), suggesting that the activation of the PI3K/AKT pathway is responsible for TNFα-mediated Snail stabilization.

Snail is a mainly regulated by GSK-3β, a kinase located downstream of the PI3K/AKT pathway [24], [29], [30]. GSK-3β maintains an active state in dephosphorylated form. To determine whether the stabilization of Snail by TNFα is mediated by regulation of GSK-3β activity, we treated HCT116 cells with LY294002 prior to TNFα treatment, and then the expression of p-AKT, p-GSK-3β, and Snail were determined by western blotting. We found that levels of p-AKT, p-GSK-3β, and Snail were increased after TNFα treatment at 6 h, while these effects were reversed upon treating with LY294002 alone or in combination with TNFα (Fig. 5B). We next measured the time courses for GSK-3β phosphorylation and found that the expression of p-GSK-3β increased in a time-dependent manner upon TNFα stimulation in HCT116 and Caco-2 cells (Fig. 5C). These results indicated that the stabilization of Snail by TNFα is due to the inhibition of the GSK-3β activity. To further confirm our findings, we knocked down the expression of GSK-3β in HCT116 cells using specific GSK-3β si-RNA (Fig. 5D). Compared with control, down-regulation of GSK-3β markedly elevated the levels of Snail expression (Fig. 5D). However, TNFα-mediated Snail stabilization was not further elevated after knockdown of GSK-3β (Fig. 5D). Similarly, when we treated HCT116 cells with LiCl, a potent GSK-3β inhibitor, in accordance with TNFα treatment, the expressions of Snail, p-GSK-3β, and β-catenin (a protein regulated by GSK-3β) were increased (Fig. 5E). However, the up-regulation of β-catenin was not as obvious as Snail. It might be due to intracellular localizations between β-catenin and Snail are different. To verify this hypothesis, we isolated Snail and β-catenin from membrane and nuclear of HCT116 cells treated with or without TNFα. The results revealed that different to Snail, β-catenin exists at the plasma membrane, and TNFα increases the nuclear translocation of Snail and β-catenin, but does not affect membrane β-catenin (Fig. 5F). Taken together, these results demonstrated that TNFα up-regulated Snail in HCT116 cells by activating AKT signaling that lead to the phosphorylation of GSK-3β and subsequently stabilize Snail.

### TNFα Suppresses Ubiquitylation of Snail by Inhibiting the Association of Snail and GSK-3β

**Figure 6:**
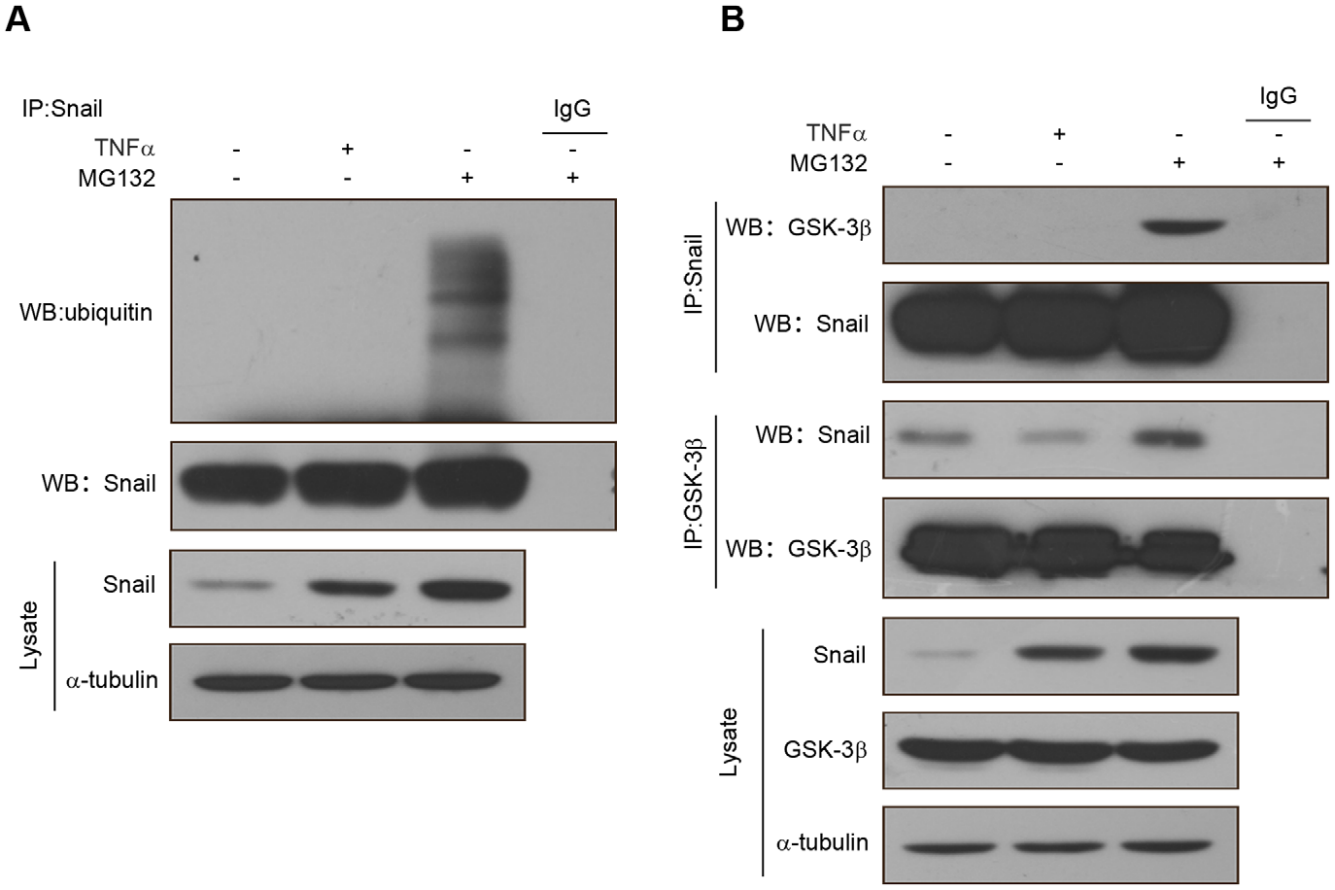
TNFα inhibits the association of Snail and GSK3β. (A) HCT116 cells were treated with TNFα (20 ng/ml) or MG132 (10 µM) for 6 h. After Snail was immunoprecipitated from equal amount of lysates (two lower panels), the ubiquitination of Snail was examined by western blotting. (B) HCT116 cells were treated with TNFα (20 ng/ml) or MG132 (10 µM) for 6 h. Snail or GSK-3β were immunoprecipitated respectively from equal amount of lysates and the associated GSK-3β or Snail were detected by western blotting.

Because the protein stability of Snail is regulated via ubiquitin-mediated proteasomal degradation processes, we speculated whether the stabilization of Snail by TNFα is mediated by suppression of Snail ubiquitylation. To test this hypothesis, HCT116 cells were treated with TNFα or the proteasome inhibitor MG132 for 6 h, and then Snail was immunoprecipitated from equal amount of lysates. The ubiquitination state of Snail was detected by western blotting with an anti-ubiquitin antibody. The results revealed that compared with MG132, TNFα dramatically suppressed the ubiquitylation of Snail, although total stabilized Snail proteins were parallel (Fig. 6A). Since GSK-3β is the main kinase that phosphorylates Snail and then induces the protein degradation of Snail [24], we next examined the association of Snail and GSK-3β. HCT116 cells were treated with TNFα or MG132 for 6 h, and then Snail was immunoprecipitated from equal amount of lysates, and the associated GSK-3β was measured by western blotting. As shown in Fig. 6B, the association of Snail with GSK-3β was diminished in cells treated with TNFα, compared with cells treated with MG-132. Similarly, when GSK-3β was immunoprecipitated from HCT116 cells, the associated Snail was markedly decreased in cells treated with TNFα, compared with cells treated with MG-132 (Fig. 6B). Taken together, these findings demonstrated that TNFα inhibited the association of Snail with GSK-3β and subsequently suppressed ubiquitylation of Snail.

## Discussion

A major challenge during cancer therapy is metastasis induced by chronic inflammation. However, the underlying mechanisms are not entirely illustrated. Several inflammatory mediators, such as TGFβ and IL-6, have been demonstrated that contribute to the invasion and metastasis of cancers [3]. TNFα is a major pro-inflammatory cytokine which has a wide range of biological activities, including inflammation, apoptosis, cell proliferation and differentiation [31]. Although TNFα has been considered as an anticancer agent, it is currently recognized that chronically elevated TNFα in tissues may promote tumor growth, invasion and metastasis [32]. There are some reports that TNFα expression is increased in the serum of CRC patients [33]. TNFα expression is also associated with tumor progression of colorectal adenocarcinomas [34]. In addition, High TNFα expression is strongly associated with tumor recurrence in CRC patients with positive lymph node metastase [34]. TNFα may be useful as a maker for the early diagnosis of CRC. In this study, we investigated an important signaling axis that controls inflammatory cytokines and induces EMT. Despite the essential role of NF-κB in inflammatory processes, we demonstrated that AKT/GSK-3β-mediated stabilization of Snail is required for TNFα-induced EMT in CRC cells. Based on our findings, we proposed a model in which TNFα up-regulates Snail via activation of AKT signaling and thereby inhibits GSK-3β activity. TNFα also inhibits the association of Snail and GSK-3β. And then TNFα stabilized Snail transfer into the nucleus, decreases epithelial makers E-cadherin and ZO-1 expressions, increases mesenchymal makers N-cadherin and fibronectin expressions, finally induces EMT and promotes tumor metastasis.

EMT has been considered as the first step of tumor invasion and metastasis. Recent studies revealed that the expression profiles of EMT are correlated with tumor grades and metastasis of breast carcinoma [35], [36]. Also, EMT plays a pivotal role in the metastasis of colon carcinoma, which occurs at the invasive front of colon carcinoma concomitant with a selective loss of basement membrane [37]. Snail is a zinc-finger transcription factor that has been known as an essential player in the aggressive phenotype of EMT [10]. In CRC tissues, Snail was highly expressed and the inverse correlation between Snail and E-cadherin was observed. Moreover, aberrant Snail expression correlated significantly with lymph node metastasis of CRC [38]. A recent study showed that TNFα induces EMT via up-regulation of Twist in breast cancer cells [39]. TNFα can also up-regulate Slug, which imparts breast cancer cells with a stem cell-like phenotype [40]. In this study, we detected the protein and mRNA levels of Snail, Slug, Twist and ZEB1 in HCT116 cells after exposure to TNFα. The protein level of Snail but not mRNA level exhibited a rapid response to TNFα stimulation. However, TNFα treatment altered neither mRNA nor protein levels of ZEB1, Slug and Twist. Overexpressed Snail also induces EMT, indicating that Snail may initiate EMT in TNFα-treated HCT116 cells. Accordingly, gene silencing of Snail by si-RNA abolished TNFα-induced morphological changes and partly recovered the TNFα down regulated E-cadherin expression in HCT116 cells. These results suggested that there is a functional linkage between Snail expression and TNFα-mediated EMT in HCT116 cells. The knockdown of Snail did not fully reverse EMT induced by TNFα. Previous studies have demonstrated the function of many other transcription factors, such as ZEB2, E47, in controlling EMT during cancer progression [14]. It is worth to further investigating whether Snail can cooperate with the other transcription factors for the regulation of TNFα-mediated EMT in HCT116 cells.

Snail is a highly unstable protein with a short half-life and is regulated by a complex signaling network at both the transcriptional and post-transcriptional levels [41]. In this study, we demonstrated that TNFα stabilizes Snail via post-transcriptional regulation processes. A body of studies pointed out that NF-κB signaling is a crucial mediator linking between inflammation and cancer [42]. For example, NF-κB-mediated up-regulation of Twist is required for TNFα-induced EMT of breast cancer [39]. A recent study further demonstrated that inflammation induces invasion and metastasis via NF-κB-mediated stabilization of Snail [43]. They found that NF-κB inhibitor suppresses TNFα-stabilized Snail in Snail/HEK293 cells. However, our observations showed that in CRC cells NF-κB is not crucial for stabilization of Snail mediated by TNFα. The TNFα-enhanced Snail was blocked by treatment with PI3K inhibitor, but not NF-κB inhibitor, suggesting that PI3K/AKT signaling pathway plays an essential role for TNFα-stabilized Snail of CRC cells. AKT pathway is frequently activated in various cancers and plays an critical role in promoting EMT and invasion [44]–[47]. For instance, activation of AKT pathway is required for induction of TGFβ- and EGF-dependent EMT [46]. AKT can also phosphorylate IKKα to increase Snail expression and induce EMT [48]. To date, GSK-3β has been characterized as a main kinase responsible for the subcellular location and protein stability of Snail [24], [29], [30]. In the present work, we found that activation of upstream AKT signaling represses GSK-3β activity. Also TNFα inhibits the association of Snail with GSK-3β and thereby increases the stability of Snail. After knockdown of GSK-3β, TNFα-mediated Snail stabilization is not further elevated.

Besides Snail, GSK-3β regulates stability of β-catenin by phosphorylating serine or threonine residues in its N-terminal domain [49]. In our study, we found that β-catenin was up-regulated by TNFαand LiCl (Figure 5E). However, this up-regulation was not as obvious as Snail. The reason may due to the different intracellular localizations between β-catenin and Snail. Though Snail and β-catenin localize in both the cytoplasm and the nucleus, β-catenin acts as an important component of the action cytoskeleton at the plasma membrane [50]. Only cytosolic β-catenin can be phosphorylated by GSK-3-β [49], so the alteration of β-catenin in whole cell lysate is not distinct with TNFα treatment.

In summary, we demonstrated that Snail plays a critical role in TNFα-induced EMT in HCT16 and Caco-2 cells. We also showed that TNFα up-regulates Snail by increasing its stability in two ways. On the one hand, TNFα activates AKT signaling and subsequently represses GSK-3β activity. On the other hand, TNFα inhibits the association of Snail with GSK-3β. These discoveries may provide a better understanding for colorectal cancer signaling and introduce potential therapeutic targets for malignant colorectal cancer.
